# Designing an Herbarium Digitisation Workflow with Built-In Image Quality Management

**DOI:** 10.3897/BDJ.8.e47051

**Published:** 2020-03-26

**Authors:** Abraham Nieva de la Hidalga, Paul L Rosin, Xianfang Sun, Ann Bogaerts, Niko De Meeter, Sofie De Smedt, Maarten Strack van Schijndel, Paul Van Wambeke, Quentin Groom

**Affiliations:** 1 School of Computer Science and Informatics - Cardiff University, Cardiff, United Kingdom School of Computer Science and Informatics - Cardiff University Cardiff United Kingdom; 2 Meise Botanic Garden, Meise, Belgium Meise Botanic Garden Meise Belgium

**Keywords:** Data capture, digitisation workflow, image quality control, herbarium sheets, natural history collections, digital specimen

## Abstract

Digitisation of natural history collections has evolved from creating databases for the recording of specimens’ catalogue and label data to include digital images of specimens. This has been driven by several important factors, such as a need to increase global accessibility to specimens and to preserve the original specimens by limiting their manual handling. The size of the collections pointed to the need of high throughput digitisation workflows. However, digital imaging of large numbers of fragile specimens is an expensive and time-consuming process that should be performed only once. To achieve this, the digital images produced need to be useful for the largest set of applications possible and have a potentially unlimited shelf life. The constraints on digitisation speed need to be balanced against the applicability and longevity of the images, which, in turn, depend directly on the quality of those images. As a result, the quality criteria that specimen images need to fulfil influence the design, implementation and execution of digitisation workflows. Different standards and guidelines for producing quality research images from specimens have been proposed; however, their actual adaptation to suit the needs of different types of specimens requires further analysis. This paper presents the digitisation workflow implemented by Meise Botanic Garden (MBG). This workflow is relevant because of its modular design, its strong focus on image quality assessment, its flexibility that allows combining in-house and outsourced digitisation, processing, preservation and publishing facilities and its capacity to evolve for integrating alternative components from different sources. The design and operation of the digitisation workflow is provided to showcase how it was derived, with particular attention to the built-in audit trail within the workflow, which ensures the scalable production of high-quality specimen images and how this audit trail ensures that new modules do not affect either the speed of imaging or the quality of the images produced.

## Introduction

Digital imaging of large numbers of fragile specimens is an expensive and time-consuming process that is likely to be done only once. Consequently, the digital images produced need to be useful for the largest set of applications possible and have a potentially infinite shelf life (in theory). The applicability and longevity of the images depend directly on their quality. As a result, the quality criteria that specimen images need to fulfil must influence the design, implementation and execution of the digitisation workflows. All aspects of the workflow are affected, including selection of equipment, definition of image and data formats, digital curation practices, image processing software and definition of operational constraints. In response to this challenge, the digitisation team at Meise Botanic Garden (MBG) has designed, implemented and operated a modular digitisation workflow which can support in-house and outsourced digitisation campaigns. The workflow is operated normally to support the continuous digitisation of specimens using in-house facilities handling hundreds of specimens per day. However, it can scale-up to support mass digitisation campaigns which process thousands of specimens daily. This article reports on the design and operation of the digitisation workflow and is provided to showcase how this workflow was designed and implemented, particularly looking at the built-in audit trail within the workflow, which ensures the production of high-quality specimen images. Mass digitisation of the world’s specimens is required for two main reasons. Firstly, digitisation can provide a permanent record of a specimen even if the original eventually deteriorates or becomes unavailable (lost or destroyed). Digitisation also reduces wear on specimens because they are not handled manually every time they are consulted. Secondly, perhaps more importantly, digitisation will significantly enhance the accessibility of specimens. For many applications, a digital image of a specimen can replace the original, with the added benefits of being endlessly shared, duplicated, edited and printed. Accordingly, a fully digitised herbarium is a useful research tool for scientists, not only locally, but also globally. For example, digitisation can be relevant for countries in the tropics and southern hemisphere, given that many important collections from those countries are held in institutions located in Europe and North America. Considering that the ideal is to digitise collections once, it is critical that the digitisation process does not limit the eventual uses of the images. Potential applications include basic ones, such as determining the identity of the specimen and reading the label details but they might also include automated extraction of character traits using pattern recognition or information extraction from labels and annotations through optical character recognition (Corney et al. 2012, Drinkwater et al. 2014, Corney et al. 2018). More advanced uses of images demand higher quality images and the increased usefulness must be balanced against the additional costs of capture and storage. In recent years, digital storage costs have reduced, but the long-term, year-on-year, costs of storing large quantities of digital images in a system from where they can be quickly and reliably retrieved are still substantial. For instance, MBG has an agreement with the Flemish Institute for Archiving (Vlaams Instituut voor Archivering - VIAA) for long term preservation which costs €120.00 (VAT excl.) per TB per year. In comparison, the commercial providers storage offerings range from €19.60 to €239.60, depending on the type of access required*^4^. There are also additional costs for handling of large data volumes, because larger images take longer to process, to convert into different formats and to transfer over networks. For these reasons, a compromise must be made between the desire to store the highest quality specimen images and the costs of creating, storing and managing those images.

Specimen digitisation workflows run at different paces depending on the degree of automation, type of collection and specimen handling protocols (e.g. Allan et al. 2019, Barber et al. 2013, Granzow-de la Cerda and Beach 2010, [Bibr B5316728][Bibr B5475780]). With the goal of producing high quality digital specimens, the digitisation team at MBG designed a flexible digitisation workflow which integrates quality management activities aligned with variable digitisation rates. The integration was designed to be transparent, with negligible impact on workflow throughput. The purposeful design of quality management activities allows fine tuning, preventing situations in which quality control activities are conducted too slowly or too infrequently, thereby increasing the risk of quality lapses, late discovery and a waste of resources. The success of collection digitisation campaigns depends directly on the fitness for use of the digital specimens. As a result, quality management activities are essential to guarantee the usefulness of digital images and should be planned and developed accordingly. Insufficient consideration of the time and effort required for quality management can potentially waste the investment in digitisation and would have long term consequences.

The paper is structured as follows. The first section describes the context in which the original workflow was implemented and the drivers for modularising and extending the workflow to allow the participation of external providers, as well as the quality criteria observed during its design and evolution. The second section describes the digitisation workflow, describing the tasks performed, the actors participating in its execution and the products derived from it. The third section elaborates on the implementation of image quality management within the workflow, by describing the images' audit trail. The fourth section discusses the results obtained with the operation of the MBG workflow and compares it to similar efforts. Finally, the fifth section describes the conclusions and further work.

## Context

The world’s 3,095 active herbaria contain an estimated 387 million specimens (Thiers 2018). These research objects are an invaluable, irreplaceable resource for science. Their taxonomic and nomenclatural usefulness is obvious, but they are also used for research into biogeography, evolution, ecology and climate change (MacGillivray et al. 2009, Schuettpelz et al. 2017, Kho et al. 2018, Corney et al. 2018, Vissers et al. 2017). In recent years, biological collections have begun to digitally image their specimens. This has been driven by several important factors, but include a need to increase global accessibility to these specimens and to help preserve the original specimens, by reducing their handling. Digitisation enables a dispersed global workforce of biodiversity scientists to access these primary data (Baird 2010).

Until 2015, MBG had digitised over 100,000 specimens, mainly funded by the Andrew W. Mellon Foundation’s Global Plants Initiative (Royal Botanic Gardens Kew 2019, JSTOR 2018a, JSTOR 2018b). This in-house digitisation process was based on two scanner-based digitisation stations, an internal network of servers for storage, processing, archiving and publishing, a custom digitisation workflow and a team of operators and IT support personnel working closely with curators and volunteers at MBG. However, the herbarium contains about 4 million specimens and a significant scaling-up of the digitisation effort was required to complete the process. In 2015, MBG received a grant from the Flemish Government to digitise a set of 1.2 million vascular plants specimens, particularly all those from Belgium and Africa (Meise Botanic Garden 2015). The grant was also to fund the infrastructure required to support the ongoing digitisation capacity after the end of the project. Scaling up the digitisation process, while preserving the high quality of the images produced, required subcontracting two parts of the workflow: the digitisation of specimens and the archiving of high definition images. Meanwhile, the internal digitisation team would concentrate on verifying the quality of the images produced, as well as on the pre-digitisation, image curation (information extraction and annotation) and publishing.

### Quality Criteria

The definition of quality criteria can serve to manage the expectations of digitisation processes, guide the acquisition of equipment, the selection of processing software and the selection of storage and publishing infrastructures. This section describes recommended quality criteria of research quality images. The criteria are derived from practical experience of the MBG while implementing and improving their digitisation practices, in line with established standards and recommendations.

The Global Plants Initiative guideline for herbarium specimen digitisation (JSTOR 2018a, JSTOR 2018b) establishes 600 PPI as the recommended resolution for scanning herbarium sheets. This resolution applies to the TIFF image. This image is not usually published but serves as the base from which other images versions are derived. The resolution of these images will vary according to the intended use of the derived images (such as web publishing or printing).

Table 1 describes the standard criteria for digital images of herbarium sheets. The details about the different resolutions and uses are derived from the recommendation from the Library of Congress (Library of Congress 2018), Synthesys 3 (Phillips et al. 2014), Global Plants Initiative (JSTOR 2018a, JSTOR 2018b) and the Federal Agencies Digitisation Guidelines (Federal Agencies Digital Guidelines Initiative 2016, Federal Agencies Digital Guidelines Initiative 2018). The recommendations for bit depth and colour accuracy are derived from the technical recommendation from the Library of Congress (LoC 2018, Library of Congress 2018). Finally, the recommended colour space is Adobe RGB (1998), taken from FADGI (Federal Agencies Digital Guidelines Initiative 2016, Federal Agencies Digital Guidelines Initiative 2018). The quality management criteria can be used to implement quality assessment activities within the digitisation workflow.

MBG determined that, for every specimen, a set of three images need to be produced: a high definition uncompressed archive quality master image (TIFF 450 PPI). Apart from the master image, two derivatives are produced: a high definition lossless image for derivation of other images (JPEG2000 420 PPI) and a lossy image for web publishing/online inspection (JPEG 420 PPI). The high definition uncompressed image for archiving is intended for long-time preservation, the high definition lossless image is intended to provide a working image which is easy to store, transfer and process and the low-resolution image is intended for online publishing. Any other image derivatives required can be produced as needed.

The DPI value is largely meaningless, because it relates to an arbitrary print size of the object. The Global Plants Initiative (GPI) project (Royal Botanic Gardens Kew 2019, JSTOR 2018a, JSTOR 2018b) specification of 600 PPI resolution is linked to the use of flatbed scanners, which were the main type of digitisation equipment when the recommendation was produced (between 2004 and 2009). The 600 PPI related to the highest resolution of the scanner sensor which still allowed fast digitisation and good quality reproduction, assuming that the print size of the image would be the same as the original specimen size. The 450 PPI value comes from assuming a print size where 450 camera pixel lengths would be printed in one inch of paper. With the high density sensors in the modern camera, the print size to achieve 450 ppi would be very large. Therefore, the PPI resolutions are relative to assuming different final print sizes. The camera actually has a sensor with a pixel density of about 4 MP/cm², which is many times the resolution of flatbed scanners.

### Additional criteria

**Figure 1.**
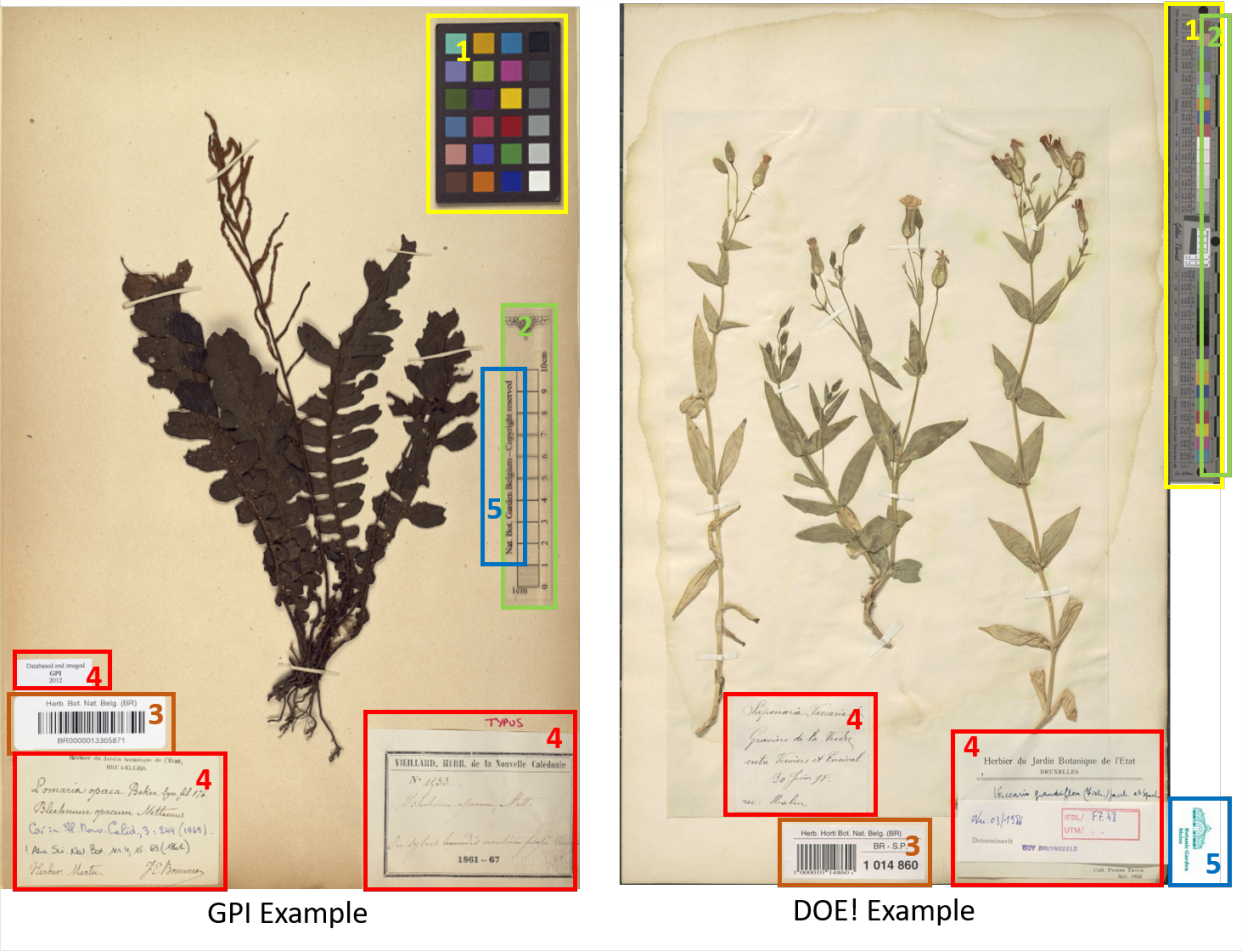
Examples of herbarium sheets and the required elements to capture. The left image corresponds to a specimen digitised during the GPI project and the one on the right is an specimen digitised during DOE!. The elements are (1) Colour Chart, (2) Scale Bar, (3) Barcode, (4) Labels and (5) Institution Name. As the images show, some elements may be combined, for instance the scale bar and institution name on the left and colour chart and scale on the right.*^1^

In addition to the image quality requirements described above, herbarium sheet images must include a set of image elements. Image elements refer to visual elements which appear next to the herbarium sheet specimen and which are intended to help in the identification, processing and quality control. There are five elements recommended by the Global Plants Initiative (JSTOR 2018a, JSTOR 2018b): (1) Colour Chart, (2) Scale Bar, (3) Labels, (4) Barcode and (5) Institution Name. In the case of herbarium sheets, imaging all the elements may, on occasion, require more than one pass, since they may be in the form of booklets or paper sheets attached to the herbarium specimen. Fig. 1 shows the elements of the herbarium sheet specimen.

The **colour chart** is recommended for helping with quality control and post-processing; this can help in verifying the lighting, white balance and colour accuracy of the image. The Federal Agencies Guideline for Digitisation, the Library of Congress and Synthesys 3, recommend the use of the colour chart, referenced as colour target or colour checker (Federal Agencies Digital Guidelines Initiative 2016, JSTOR 2018a, JSTOR 2018b, Library of Congress 2018, Phillips et al. 2014). There are many types of colour charts and examples of many of them have been used by different institutions in their digitisation workflows. However, modern targets, such as those from Image Science Associates, are preferred over legacy targets (Colour Control Patches from Kodak), because they were developed for digital image creation and are made to tight tolerances (Cultural Heritage, Digital Transitions Division of Cultural Heritage 2018, Image Science Associates 2017). Object level targets of this type include a ruler and can be used for verification of colour, sharpness and scale.

**Scale bar** is recommended to enable the calculation of the dimensions of the specimen (Phillips et al. 2014).

**Herbarium name** (with or without logo) is required to quickly identify the institution holding the specimen (Phillips et al. 2014).

**Labels** are commonly placed next to the specimen attached to the herbarium sheet. Clear capture of labels is important for further processing and documentation of the specimens (Phillips et al. 2014).

**Barcodes** are identifiers used for cataloguing specimens which are also useful for linking them to digital specimens. Synthesys 3 and GPI recommend the use of barcodes as internal identifiers which are important for further documentation and linking of the physical and digital specimens (JSTOR 2018a, JSTOR 2018b, Phillips et al. 2014). There are different types of barcodes available. Line Barcodes (one dimensional), like the ones shown on Figure 1, have been used in MBG projects. Some guidelines recommend the use of two-dimensional barcodes (Zaman 2015) to prevent misidentification; however, MBG did not use these because they are not always readable without a scanner and curators also need to be able to use them for identification of physical specimens.

## Digitisation workflow

The MBG workflow is designed to handle in-house and outsourced imaging. The in-house mode of the workflow is run entirely by MBG, while the outsourced mode is designed to seamlessly integrate the outputs from a digitisation line run by an external contractor, so it is managed jointly by the contractors and MBG. When operating in internal mode, the MBG digitisation workflow can produce images at a rate of 5000 image sets*^2^ per week using two manual camera-based digitisation stations. In contrast, the contracted digitisation line has a capability of producing 25,000 image sets per week. This throughput is determined by the ingestion capacity of the MBG in-house systems which process images through quality control, storage and publishing.

**Figure 2.**
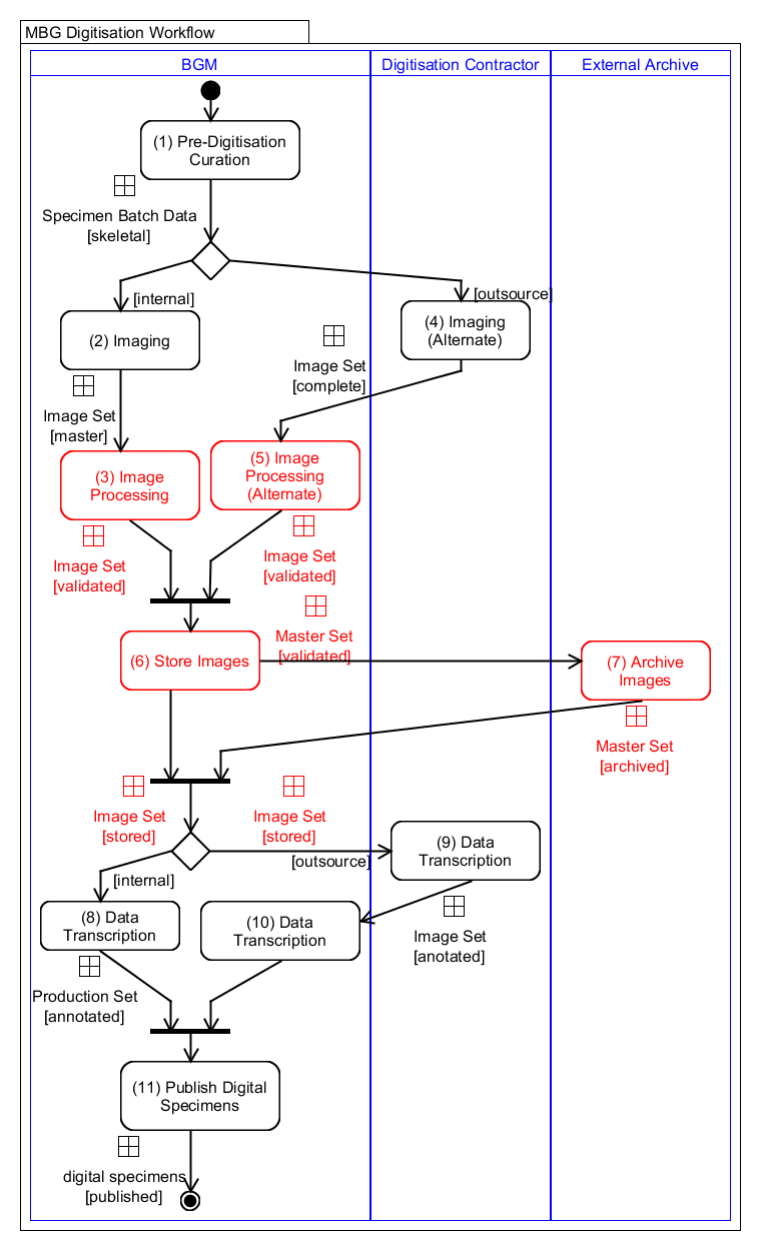
MBG digitisation workflow diagram. The circle shapes at the top and bottom indicate the start and end of the workflow. The rounded corner boxes represent workflow tasks (described in Table 2). The lines connecting tasks indicate flow of execution. The squares on connecting lines represent the data objects produced. The diamond shapes indicate a fork or merge of the flow. The bar shapes represent flow synchronisation, i.e. processing waits for completion of previous tasks.

The workflow consists of eleven tasks performed during digitisation. Table 2 provides a description of the workflow tasks and their influence in the quality management of images. The first column presents the list of tasks in order of execution, indicating in brackets the alternate tasks. The second column indicates the sub-tasks comprising each of the main tasks. Finally, the third column, indicates the quality management considerations which influence the quality of the images produced. Fig. 2 presents a diagram of the workflow including the alternative flows, actors and resources.

The final output from this digitisation process is not a set of records, it is a set of digital specimens. The digital specimen concept is intended to define a representation (digital object) that brings together an array of heterogeneous data types, which are themselves alternative physical specimen representations. In this case, the digital specimen (DS) holds references to specimen data from a collection management system, images, 3D models, research articles, DNA sequences, collector information, amongst many other data types ([Bibr B5476278][Bibr B5476268]). In this context, the output from the workflow is a collection of digital objects, which are formed of data, metadata and image files which are consistently interlinked and coherent into an entity called digital specimen.

Pre-digitisation curation (1), Image storage (6), Archive Image (7) and Digital Specimen Publishing (11) are performed during both internal and outsourced digitisation, while the Imaging (2), Image Processing (3), Imaging Alternate (4) and Image Processing Alternate (5), Data Transcription (8), Data Transcription Alternate (9) and Data Transcription Validation Alternate (10) vary depending on the decision to perform imaging internally or outsource it. The main difference is that, in outsourced mode, the contractor digitises the specimens and produces the image derivatives. For this reason, there are fewer sub-tasks to perform as part of the processing image (alternate). The differences between these tasks will be further analysed in the following section as the details of the variation becomes clearer when describing the quality management activities.

Fig. 2 shows an activity diagram of the two workflow configurations. The outer boxes (swim-lanes) indicate the entity performing a set of tasks. The filled circle at the top indicates the start of the workflow (entry point) and the smaller filled circle with double outline at the bottom indicates the end of the workflow (exit point). The diagram provides an overview of the workflow execution and the associated data lifecycle. The diagram also presents the three entities executing the workflow: MBG, digitisation contractor and external archive. Integrating image quality management activities in the MBG digitisation workflow was an early design decision that required defining the sub-tasks during which quality assessment activities are performed, the criteria to follow and the structure of the provenance chain. The execution of the quality management (QM) process within the MBG workflow is called the audit trail. This is explained in greater detail in the next section.

## Audit Trail: Integrating image quality management in the digitisation workflow

Quality management methods can be subdivided into two main areas: Quality Assurance (QA) and Quality Control (QC) (Drob 2013, Thakur 2010). QA activities are performed within the production processes to ensure that products or services are produced/delivered according to a predefined set of quality criteria. QC activities are performed after the production processes to verify that finished products or services (outputs) conform to the established quality criteria. In line with these definitions, in the MBG workflow, QA activities are implemented to verify images in a given set while being produced (acquired, derived, copied), while QC activities are implemented to verify sets of images produced externally (usually verifying a subset of the images). The QA and QC activities are implemented in the image processing and image storage tasks of the MBG workflow (3, 5 and 6 in Table 2). Quality management activities are implemented in these tasks because images are generated or (potentially) altered in some way (i.e. processing, generation of derivatives and transferring). The input for the image processing task is a batch of images, which may be created over a day or a week, as well as a metadata file containing basic image information (filename, time, operator, batch number, MD5 hash value, digitisation station). The procedure for tracing the evolution of the image sets as they are validated and transformed before publishing is designated the audit trail. The audit trail is devised to ensure that no images are lost and that they can be traced through each processing step. This ensures that every image in the batch has passed quality management, is archived and is ready for publishing in the portal. When the digitisation task is outsourced, the audit trail is important as a means to verify that the datasets delivered meet contractual agreements. MBG established a dual control system for managing the audit trail. The system consists of a database for storing image processing metadata for each sub-task. Simultaneously, operators overseeing the processing and storage tasks have access to a shared template spreadsheet where they register the advance in processing a batch. This dual control system enables work in parallel and provides an up-to-date view of the processing for operators, technicians and managers. Additionally, a designated quality control manager tracks each batch progress. The role of quality control manager can be assigned to different operators, since all members of the image processing team understand the audit trail management process.

Table 3 and Table 4 present descriptions of the sub-tasks performed as part of the image processing and store image tasks, respectively.

The following subsections will elaborate on the description and technical details of the sub-tasks that are directly related to quality management. The sub-tasks are presented in order of occurrence in the workflow. Additionally, each subsection includes a table with the technical details of each sub-task describing the agent that performs the sub-task, a brief description of the sub-task, the dependencies of the sub-task (required software, hardware) and the target entity of the sub-task (the specific datasets to be affected). This organisation is specifically designed to allow, in future, mapping the sub-tasks of the audit trail with a standard provenance model (such as PROV-O W3C 2013).

### Check file name

In the MBG collection, herbarium sheets specimens are identified by a barcode label. These labels conform either to the UPC-A or Code 128 format. In line with current practice in herbarium management (JSTOR 2018b, Phillips et al. 2014, Royal Botanic Gardens Kew 2019), the digital image files of specimens are assigned filenames based on the barcode. The check file name sub-task relies on a script that uses the ZBAR (Brown 2010) open source library to read the barcodes. The technical details of this sub-task are summarised in Table 5. The script verifies that each name conforms to MBG barcode conventions (length, structure, file type). The script works correctly for most of the images processed. However, there are cases in which two or more specimens and their barcodes are fixed to the same herbarium sheet. These cases may be flagged as incorrectly named and need to be verified manually. ZBAR can detect multiple barcodes, but a manual step is required to duplicate the image files and rename them, so that there is one image for each specimen/barcode.

At MBG, curators follow the recommendation of placing barcodes as close to the bottom right corner of the sheet as possible. If the location of the barcode is known beforehand, ZBAR can be configured to read just that part of the image, speeding up processing time considerably. However, this would only work in a collection where there cannot be two specimens on the same sheet.

### Checking file size, image dimensions and resolution

Most herbarium sheets have a standard size. Consequently, herbarium sheet imaging produces images of a size which fall within a predictable range. Based on this observation, MBG established a heuristic rule correlating file size, cropping and image resolution. This has helped in establishing the accepted file size limits for the different types of image files handled and generated during image processing. The technical details of this sub-task are summarised in Table 6.

This process also includes verifying the width and height of the image which is also a dependable indicator for detecting bad crops and malformed images. The check file size and resolution sub-task relies on a script that used statistical data of past digitisation campaigns. The script collects the size of each image file and verifies that it falls within the expected range. Images outside this range are flagged as examples of bad cropping. Large files tend to be under-cropped and small files over-cropped. This automated size check can be relied on to test cropping on a large image set, but it is less sensitive than a visual cropping check. A visual cropping check can identify small cropping problems on images, but it is impractical for large datasets. Similarly, the resolution of the image in pixels is related to the file size. In a previous version, the script tested the resolution of the image in dots per inch (DPI), because it was part of the contract terms with the external contractors. However, testing the dimensions of the image in pixels is a simpler reliable test of resolution.

### Check TIFF metadata file structure

The MD5 algorithm is a hash function used to create a checksum for a file. It can be used to ensure that an image has not been corrupted. The hash function is created soon after the image is made and needs to be stored for later use. The check TIFF Metadata File Structure sub-task relies on a script that computes the MD5 values from the image files and verifies that it matches the value generated for the original file, shortly after digitisation (md5deep and hashdeep software packages, Table 7). Initially, validation of MD5 values was conducted on entire batch sets; however, after various digitisation campaigns in which no errors were found, the process was modified to perform spot checks on a subset of the TIFF master set, for quality control purposes. Additionally, calculation and verification of MD5 is time-consuming and applying it strategically helps speeding up the execution of the entire image processing task. In addition, the hash values are stored and passed on to the long-term archive where it is used to verify the files deposited in long-term storage. The MD5 value may be used in the future to ensure the integrity of the files in long-term storage, to verify file integrity after transmission, as well as the integrity of files which are recovered from the archive when required.

### Checking for duplicates

The large numbers of specimens being processed in mass digitisation operations increases the risk of duplicate imaging. Additionally, as the description of the Check File Name sub-task explains, when a herbarium sheet contains more than one specimen, the image file is duplicated manually as many times as needed, to produce at least one image for each barcoded specimen on it.

MBG created a script that searches and flags image duplicates. The script uses barcode verification and md5deep (Table 8) to search for potential duplicates in the image set being processed. This check is not difficult nor time-consuming if the MD5 values are available.

### Check Structure

For long-term archiving, the specimen images need to conform to well-known standards, in order to improve the chances of recovery in the future. The Tagged Image File Format (TIFF) format is recommended standard for long-term storage (Ariño and Galicia 2005). Additionally, high definition images, which can be made available for wide use, are stored onsite. JPEG2000 is the format selected for storing production images on site (JP2). This format was selected because it allows storing compressed images that are lighter (i.e. smaller file size) than TIFF images, retains all the image information (lossless, no loss of information in the compression) and their quality does not degrade during generation or transmission. These images are designated production images as they are intended for publishing, sharing and generation of new derivatives. A third set of images for publishing and online inspection is generated in JPG format. This set is also part of the production set and it is used for publishing and inspection online. This set is not validated. MBG created a script that verifies the conformity of the images in the master set (TIFF) and those of the production set (JP2000) while searching and flagging image duplicates. For verifying that the images conform to the selected standards, the script uses JHOVE for TIFF images (Open Preservation Foundation 2019b) and jpylyzer (Open Preservation Foundation 2019a for JP2000 images (Table 9). The integrity of the derived JPEG files is not verified because they can always be recreated from the master (TIFF) or the high definition lossless (JP2000) images.

### Visual QC of TIFF files

There is still a need to visually verify a subset of the images for quality control. This is particularly important when the digitisation process has been outsourced as this is the means for verifying that the provider meets the service level agreements. Table 10 shows the technical details of this sub-task. Additional checks to verify image quality are performed visually, the quality problems verified in this sub-task being: the focus, cropping, colour, contrast and a visual check to see if the barcode is indeed corresponding with the filing name. Visual inspection is a time-consuming process and cannot be performed for every image in large datasets. For this reason, the verification is performed in a sample, in two stages. For the first 10 batches, 2% of the images are inspected (the last of every fifty images). If the tests do not detect a problem, the set is reduced to 1% of the images in the set (the last of every 100 images). Quality problems, such as bad focus or poor lighting, tend to occur at a point in time and continue in every image until the fault is corrected. Therefore, rather than selecting images randomly, inspection at regular intervals throughout the batch is more effective to identify the point at which a problem started to occur.

### Remove duplicates and bad crops

Removing potential duplicates and bad crops is a process that needs to be verified visually. When some images are flagged, the processing of a batch is not stopped, i.e. the batch is not rejected if errors are detected, unless the errors are found in more than 5% of the images. Instead, the erroneous images detected are removed from the batch before storing them locally or in the long-term repository. The details of this sub-task are shown in Table 11. This is a manual task performed using the error log produced during previous verifications. It is considered a quality assurance task because it ensures that no images flagged as erroneous are saved with the rest of the batch. The rejected images are reported to the imaging team or to the contractor, for re-imaging.

### Check if production set stored

The production set consists of a set of high definition JP2 images and a set of lightweight JPG images. During processing and validation, these sets are temporarily stored on a buffer server. Once the production set has been completely processed and validated, the operator executes a task to verify that all compliant image derivatives have been copied to the image repository before the buffer is cleared in preparation for processing the next batch (Table 12).

### Clear TIFF from buffer

The master set contains the high definition uncompressed TIFF images which are intended for long-term storage preservation and future recovery purposes. During processing and validation, this set is temporarily stored on a buffer server. Once the master set has been completely processed and validated, the operator must verify that all compliant images have been copied to the external archive repository, before the buffer is cleared in preparation for processing the next batch (Table 13). The script for clearing the TIFF set from the buffer includes a step that verifies if the external archive provider has acknowledged the reception and archiving of the image file. If digitisation is outsourced, the script will also send a signal to the contractor to announce that the batch has been processed completely, so they can also clear their temporary storage.

## Results from operation the MBG workflow and similar approaches

Initially, the MBG digitisation workflow supported the digitisation of 100,000 specimens (2.5% of the collection). The successful implementation and continuous operation of the workflow during the period from 2015-2018 has allowed digitising a further 1,300,000 specimens, raising the total number of specimens digitised to 1,400,000 (35% of the collection). Of these, 1,200,000 specimens where digitised in a mass digitisation campaign conducted over a year (2016-2017) with the collaboration of an external contractor. The other 100,000 specimens have been digitised as part of the continuous digitisation operations which have become part of MBG curation processes. The addition of external partners included the tendering and selection of an experienced digitisation company (Picturae), which supported the mass digitisation campaign. Similarly, a working relationship with VIAA (Flemish Institute for Archiving) for the long term archiving of master TIFF images was established and integrated as part of the continuous digitisation efforts. At the moment, MBG is in the midst of a second mass digitisation campaign, which will digitise an additional 1,400,000 specimens and which will get MBG closer to the target of a fully digitised herbarium, reaching 70% of the collection by 2020. The modular nature of the workflow has also allowed the testing of the inclusion of different providers. In this case, as part of the ICEDIG project, a pilot study analysed the requirements for using European Open Science Cloud infrastructures for long term storage (Dillen et al. 2019, Nieva-de-la-Hidalga et al. 2019b, Agosti et al. 2019). Additionally, further automation of workflow tasks has been analysed. This includes the use of segmentation for speeding up quality control and information extraction with optical character recognition (Nieva-de-la-Hidalga et al. 2019a, Owen et al. 2019, Barber et al. 2013).

### Image Quality

**Figure 5.**
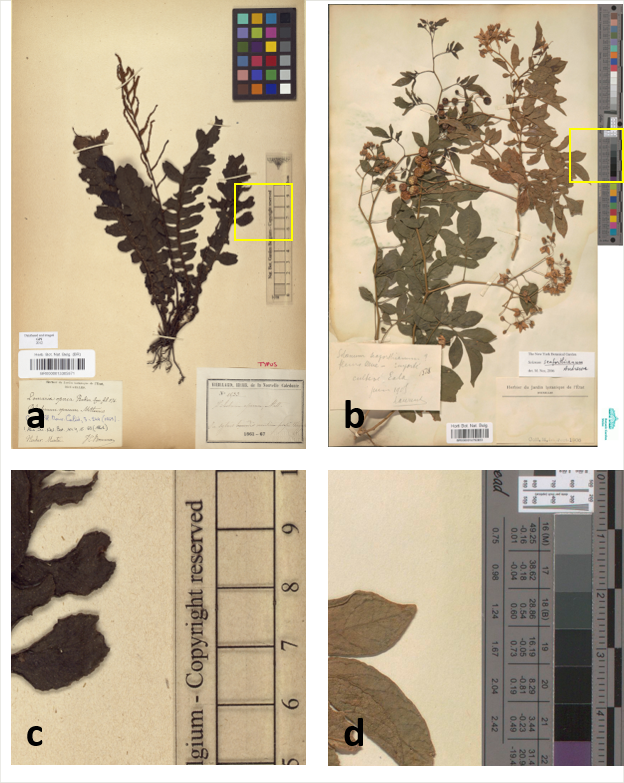
Example of results from in-house and outsourced digitisation. Image "a" corresponds to a specimen digitised in-house and image "b" corresponds to a specimen digitised by the contractor. Images "c" and "d" correspond to close-ups of the sections highlighted in "a" and "b", respectively, presented at 100% size (5x5 cm square).

The quality of the images created in-house and those outsourced are equivalent. Except for the metadata establishing which process was used for digitising, the quality criteria including image dimensions, resolution and image elements are the same. The images in Fig. 5 show examples of specimens from two herbarium sheets, digitised using in-house and outsourced digitisation, presented side by side to highlight the consistency of the results obtained. The top images (a, c) are scaled to 1/10x or 10% of their actual size. The bottom images (b, d) present 5x5 cm segments highlighted with yellow frames in "a" and "c" at a 1:1 (1x) scale. Both images were downloaded from the MBG Botanical Collections Portal (www.botanicalcollections.be) and are derivatives from the JP2000 images, encoded using the eci-RGB colour profile, 24-bit colour depth, at 420 PPI.

**Figure 6.**
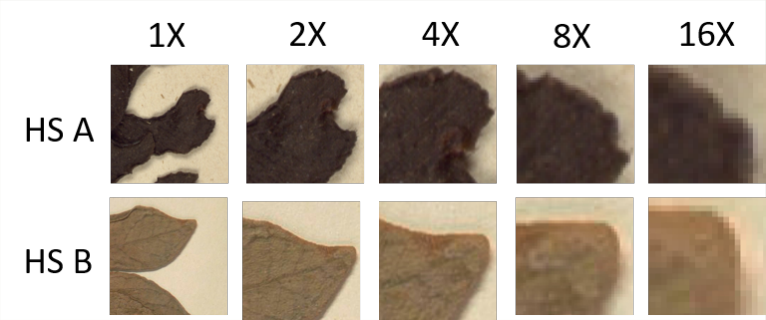
Image Resolution and Scaling Comparison. HS A fragments correspond to close-ups of the images shown in Fig. 5a-c. HS B fragments correspond to close-ups of the images shown in Fig. 5b-d.

Fig. 6 provides a further view of how the two examples from Fig. 5 compare to each other when the images are scaled. The first 1x column shows the corresponding 2x2 cm sections from Fig. 5 "b" and "d" respectively, keeping the 1:1 proportion. The remaining columns show close-up fragments of the same section scaled at 2, 4, 8 and 16 times their original size. As the images show, the magnification up to 8x is acceptable, as the borders and object features are shown clearly, with no pixelation. From 16x, the pixels are noticeable around the edges of the specimens. Magnifying physical specimens usually requires using lenses or microscopes, that only allow viewing limited sections of the specimen. However, larger high resolution displays allow viewing greater proportions of the specimen features.

### Related work

**Figure 7.**
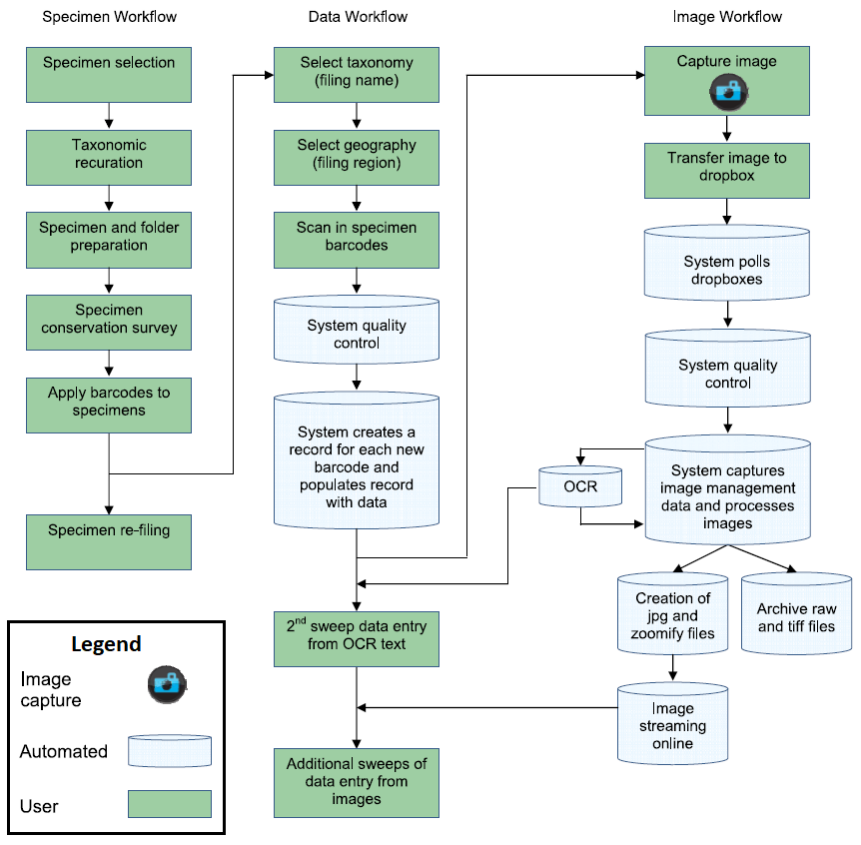
Diagram of the digitisation workflows at the Royal Botanic Garden Edinburgh (from Haston 2012).

The digitisation workflow of the Royal Botanic Garden Edinburgh (RBGE) is an example of an in-house digitisation workflow. The main reasons for designing and developing this workflow in-house was the variation of funding and scale of digitisation campaigns (Haston et al. 2012). For this, a custom and modular system which could be adapted to cater for the needs of different digitisation projects was required. The RBGE designed three concurrent workflows which responded to these requirements. Fig. 7 shows the specimen, data and image workflows side by side. Notice that the image quality control activities are part of the image workflow and occur after digitisation. The workflows developed by RBGE are suitable for the characteristics of the different funding streams and the diversity of digitisation projects. The design of modular and integrated workflows to manage the processes, images and data has served this purpose. This approach is different to the process described by MBG which includes outsourcing the mass digitisation operation and external archival partners for long term storage.

The Natural History Museum, London (NHM) has also developed a workflow for the digitisation of microscope slides, which significantly improved their digitisation efficiency, as compared to previous semi-automated methods. They also found that their automated workflow reduced the number of errors (Allan et al. 2019). Yet others have found that the modulisation of tasks in the data capture stage of processing improves efficiency and accuracy (Granzow-de la Cerda and Beach 2010).

**Figure 8.**
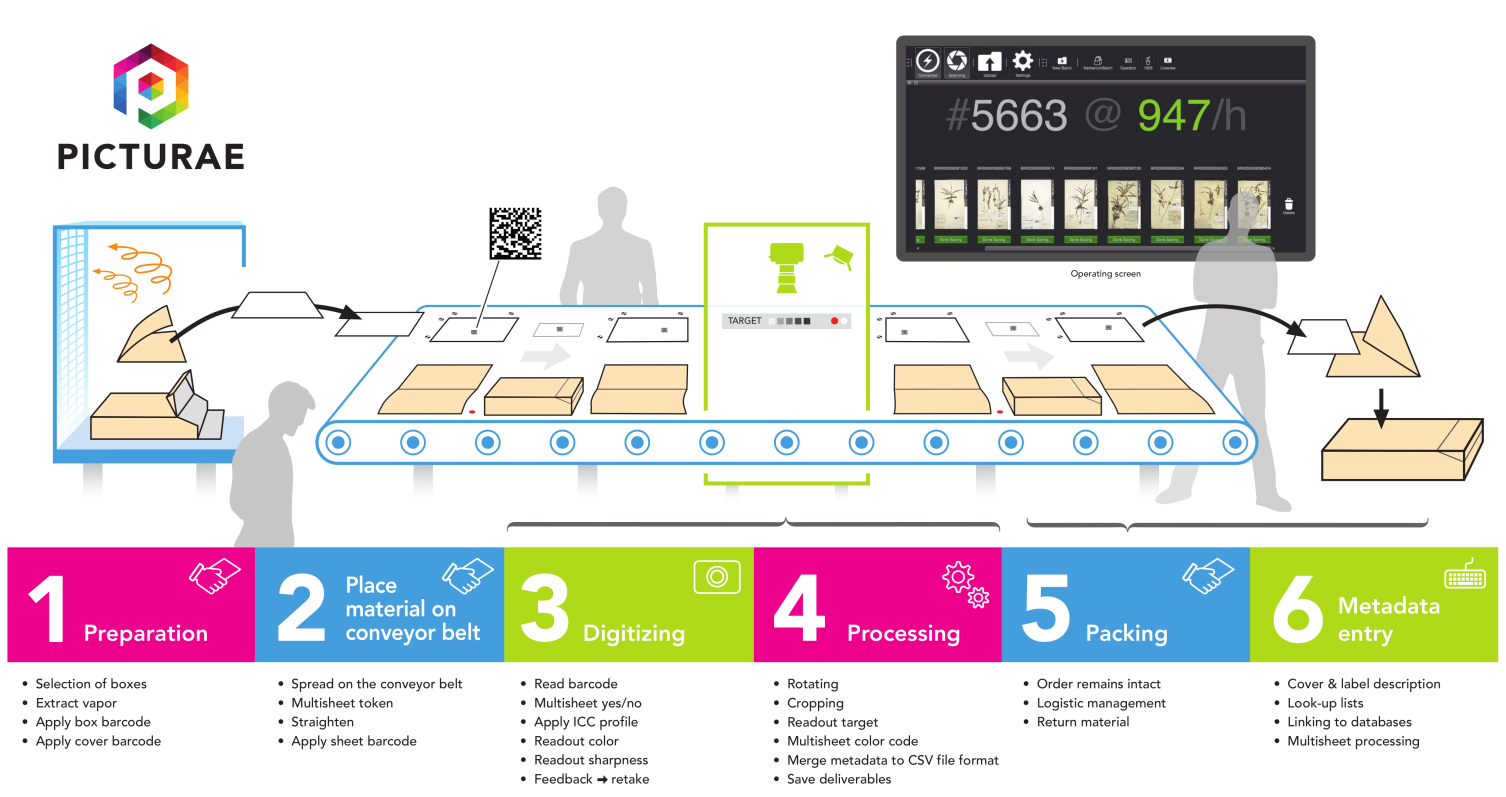
Picturae digitisation workflow for herbarium collections. The digitisation task (3) includes image quality control checks (courtesy of Picturae*^3^).

Picturae is a digitisation provider with more than ten years of experience digitising libraries and museum collections (including cultural and natural history collections). The digitisation workflows, developed by Picturae (Fig. 8), illustrate how image quality management activities are integrated (Picturae 2017). The workflow is designed to perform image quality assessment at digitisation time, stopping and rewinding the conveyor belt if an error is detected. The means for verifying quality are built into the workflow allowing the rapid verification of every image. This workflow has been applied and tested in large digitisation projects for institutions such as Naturalis (Netherlands) and the Muséum National d’histoire Naturelle (France) and in working with MBG, as reported above.

The outsourced approach is effective in mass digitisation projects. There are similar examples of such projects involving other providers, such as the digitisation of the Moscow University Herbarium (Seregin 2018) or the Natural History Museum of Norway (Digitarium 2017).

## Conclusions and future work

Image quality control is an essential aspect of the digitisation of biological collections. The imaging workflow combines physical movement of specimens with complex digital workflows and delays or acceleration of any part of the process can have negative consequences. In this workflow of many time-dependent steps, it is easy to overlook or to cut corners on quality control. However, if care is not taken at this stage, then it may impact the long-term usefulness of the images. Care needs to be taken at every stage of the process from the photography, the depositing of files on the image servers and the long-term archiving. The workflow and quality management tasks, described in this article, illustrate the process of creating high-quality digital specimen collections which are close to the ideal of the digital herbarium. The workflow tasks are constantly revised and updated to improve their speed and accuracy.

## Figures and Tables

**Figure 3.**
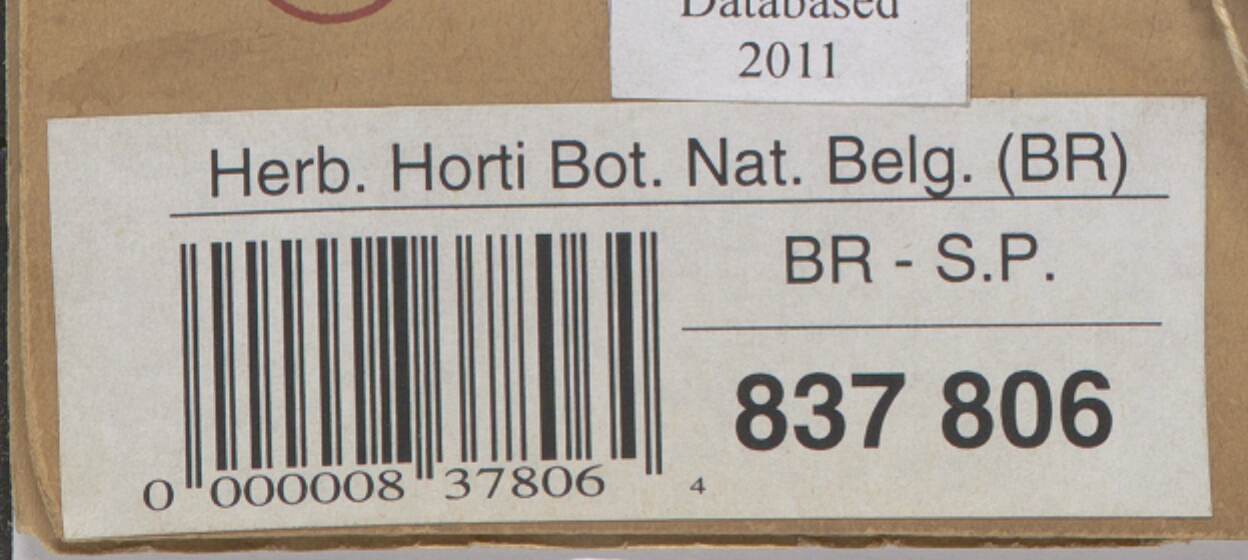
Example of an herbarium sheet barcode.

**Figure 4.**
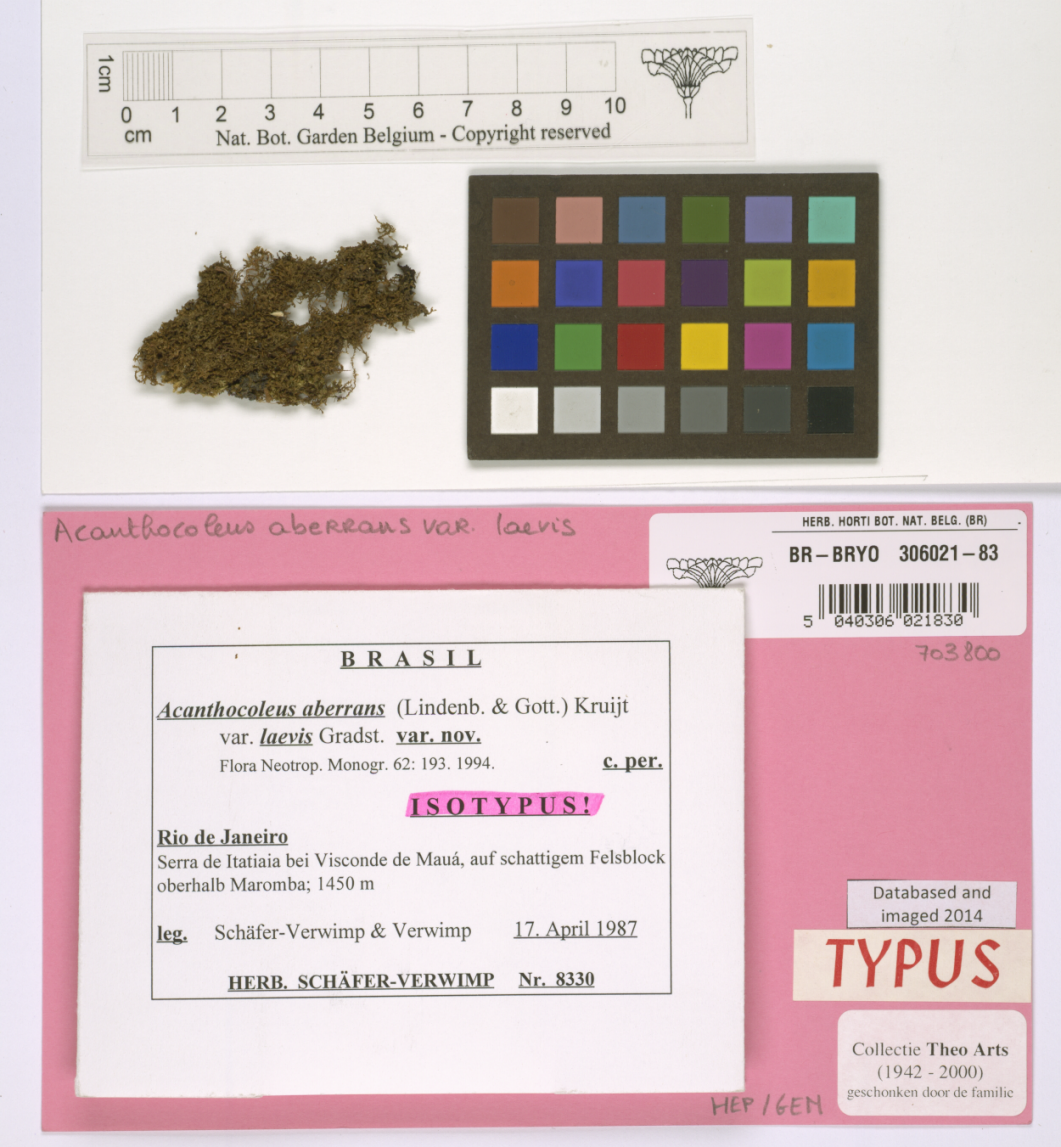
Example of a non-standard size herbarium specimen.

**Table 1.** Quality criteria for herbarium sheet images.

**Image Use**	**Resolution**	**Bit Depth**	**Grey Scale Factors**	Colour Accuracy**
Web Publishing	72 PPI	24-bit colour		ΔE < 5
Printing	300 PPI	24-bit colour		ΔE < 5
OCR Labels	400 PPI	8-bit grey scale	Min: 28 steps Min: 5.5 f-stops Y channel noise <= 5%	
Identify Specimen Features	400 PPI	24-bit colour		ΔE < 5
Research on Specimen	600 PPI*	24-bit colour		ΔE < 5
Preservation	600 PPI*	24-bit colour		ΔE < 5
* Minimum resolution recommended; if digitisation devices available allow for higher resolution, that resolution should be used. ** ΔE (Delta E, dE) is a metric for understanding how the human eye perceives colour difference. The term delta comes from mathematics, meaning change in a variable or function. The suffix E references the German word Empfindung, which broadly means sensation.				

**Table 2.** MBG digitisation workflow tasks.

**Task**	**Sub-tasks**	**Quality Concerns**
**1 Pre-digitisation curation**	Selection of specimens to digitise. Retrieval from storage. Identification of specimens (barcoding). Conservation/restoration of specimens selected for digitisation. Specifying safeguards for handling specimens. Marking specimens that are already digitised. Extraction exceptions for internal imaging (e.g. capsuled specimens or specimens that needed to be imaged twice due to added booklets). Creation of metadata record / adding cover barcodes for external transcription of the labels. Transfer to digitisation station.	Specimens are selected and prioritised for digitisation by collection curators. Some sheets may be damaged or fragile or specimens may need to be remounted to display relevant features.
**2 Imaging**	Station(s) Setup Digitisation equipment selection, acquisition and set up. Equipment testing/calibration. Training of digitisation technicians.	Equipment should be calibrated to minimise image postprocessing after digitisation.
	Digitisation Mounting for imaging Digitisation of a specimen, creation of a master file (TIFF). Unmounting and return of specimen. Data capture, based on the image when outsourced.	Identification, digitisation and [meta] data capture, so that images are correctly linked to the corresponding specimen records.
**3 Image processing**	Retrieval of master files (TIFF) from temporary storage. Creation of derivatives for publishing and distribution (JPEG2000 and JPG); Verification of naming and linking of files (based on barcode ID). Verification of file formats.	Verification of master image resolution format. Verification that derivatives adhere to quality standards.
**4 Imaging (alternate)**	Imaging (2) and image processing (3) are integrated. The Task receives specimens and produces full sets of images (TIFF, JPEG2000 and JPG).	Same as those for 2 and 3 above.
**5 Image processing (alternate)**	Verification of image sets (correspondence of master and derivatives). Verification of naming and linking of files (based on barcode ID).	The task is simpler. However, the load increases considerably, from 5,000 to 25,000 weekly specimen image sets to process (400% increase).
**6 Store images**	Transfer of master and derivative files to archive servers and image servers. Create and preserve links to storage.	Verify that master and derivative files are not corrupted in transfer to storage.
**7 Archive images**	Deposit master files on external archives for long term preservation.	Verify master is not corrupted in transfer and images are recoverable.
**8 Data transcription**	Extraction of data from images, populating/complementing specimen record. Final verification/correction of specimen data.	Verify readability of image data for transcription. Verification against reference image and recorded data before publishing.
**9 Data transcription (alternate)**	Extraction of data from images, populating/complementing specimen record.	Verify readability of image data for transcription.
**10 Data transcription validation**	Final verification/correction of specimen data.	Verification against reference image and recorded data before publishing.
**11 Publish digital specimen**	Creation of digital specimen, verifying links to images, data, physical specimen and collection management system data. Publishing of digital specimen.	Data, metadata, persistent identifiers and links are used to build stable long-lasting specimens which adhere to FAIR data principles.

**Table 3.** Image processing subtasks.

**num**	**sub-task**	**type**	**dataset**	**state**		
				**start**	**success**	**fail**
1	Check file name (Table 5)	AT, QA	TIFF set		names_ok	names_error
2	Check tiff file size, image dimensions and resolution (Table 6)	AT, QA	TIFF set	names_ok	fssr_ok	fssr_error
3	Generate JPEG 2000 derivatives	AT, IH	TIFF set	fssr_ok	jp2_gen	jp2_gen_err
			JP2 set		jp2_gen	jp2_gen_err
4	Generate jpeg derivatives	AT, IH	TIFF set	jp2_gen	jpg_gen	jpg_gen_err
			JPG set		jpg_gen	jpg_gen_err
5	Check metadata file structure (Table 7)	AT, QC	TIFF set	jpg_gen	md5_ok	md5_error
6	Check duplicates (Table 8)	AT, QA	TIFF set	md5_ok	unique	duplicate
7	Check structure and file size (Table 9)	AT, QA	TIFF set	unique	fss_ok	fss_error
			JP2 set	jp2_gen	fss_ok	fss_error
8	Visual qc tiff files (Table 10)	MT, QC	TIFF set	fss_ok	vqc_ok	vqc_error
9	Check filename (Table 5)	AT, QA, IH	JPG set	jpg_gen	jpgn_ok	jpgn_error
**Sub-task Type**: **AT** automated task, **MT** manual task, **QA** quality assurance task, **QC** quality control task, **IH** sub-task performed in-house only.						

**Table 4.** Store images sub-tasks.

**num**	**sub-task**	**type**	**dataset**	**state**		
				**start**	**success**	**fail**
1	Remove duplicates and bad crops (Table 11)	MT, QA	TIFF set	vqc_ok	dup_rmv	
			JP2 set	fss_ok	dup_rmv	
			JPG set	jpgn_ok	dup_rmv	
2	Copy files to archive	AT	JP2 set	dup_rmv	stg_ok	stg_error
			JPG set	dup_rmv	stg_ok	stg_error
3	Generate image viewers	AT	JP2 set	stg_ok	vwrg_ok	
4	Copy files to ftp server	AT	TIFF set	stg_ok	svrc_ok	svrc_error
5	Copy files to external archive	AT	TIFF set	svrc_ok	arc_ok	arc_error
6	Check jp2 and jpg sets stored (Table 12)	AT, QA	JP2 set	vwrg_ok	stgv_ok	stgv_err
			JPG set	stg_ok	stgv_ok	stgv_err
7	Clear buffer server (Table 13)	AT, QA	TIFF set	arc_ok	bufc_ok	bufc_err
8	Clear buffer server	AT	JP2 set	stgv_ok	bufc_ok	bufc_err
			JPG set	stgv_ok	bufc_ok	bufc_err
**Sub-task Type**: **AT** automated task, **MT** manual task, **QA** quality assurance task.						

**Table 5.** Check file name sub-task*.

Agent	Check-barcode (script).
Function	Verify that image file names structure is formed using the corresponding barcode.
Dependencies	ZBAR open source library for reading barcodes from image files (http://zbar.sourceforge.net/).
Target(s)	master images of TIFF set (Image Processing sub-task 1) production images on JPG and JP2 sets (Image Processing sub-task 10)
Criteria	Each file name must conform to the format: two-letter prefix (here BR) 13-digit string padded left with zeros, which contains the digits in the barcode optional suffix, 2-character underscore and letter (a-z) used if specimen is associated to more than one image (e.g. _a) file extension consistent with the set being processed (either .tif,.jp2,.jpg)
Success	Filenames are correctly formed (names_ok).
Fail	Filenames are incorrect (names_err).
Example	Valid file names for the images in the three sets corresponding to specimens with barcode from the example shown on Fig. 3. These would be: BR0000008378064.tif BR0000008378064.jp2 BR0000008378064.jpg
Exceptions	Herbarium sheets can contain more than one specimen and more than one barcode. These sheets may be flagged as incorrect and require manual processing. Additionally, herbarium sheets can have legacy barcodes from previous cataloguing efforts and, consequently, may have more than one barcode even when having only one specimen. If this is the case, the legacy barcode is removed, the image is deleted and the specimen is sent back for re-imaging.
* Owing to the need to image collections of other herbaria and various subcollections, other filename formats have had to be accommodated.	

**Table 6.** Check file size and resolution sub-task.

Agent	Check-tif-resol-and-size (script).
Function	Utilise image file size to detect resolution and cropping.
Dependencies	JHOVE: a file format identification, validation and characterisation tool (Open Preservation Foundation 2019b).
Target(s)	Master images of TIFF set (Image Processing sub-task 2).
Criteria	Each file size must be above 88 MB (average minimum file size, which is a consistent indicator of image dimensions). Additionally, the smallest and largest files of each batch are verified manually.
Success	Correct file size indicates that cropping and resolution are within the acceptable range (fssr_ok).
Fail	Incorrect file size may indicate cropping or resolution issues (fssr_err). The images need to be flagged for manual verification.
Exceptions	Some specimens can be preserved in non-standard size sheets, like the one shown on Fig. 4 which is smaller than the average herbarium sheet.

**Table 7.** Check TIFF metadata file structure sub-task.

Agent	Check-md5-meta (script).
Function	Utilise md5 checksum to verify the integrity of images after transmission, storage and recovery operations.
Dependencies	md5deep and hashdeep software packages to process verify the match between stored and computed md5 hash values (http://md5deep.sourceforge.net/).
Target(s)	Master images of TIFF set, only a subset is verified (Image Processing sub-task 5).
Criteria	Calculated md5 hashset values must coincide with stored hashset values.
Success	The image file has not changed, the copy is consistent with the original (md5_ok).
Fail	The image file has been corrupted since its creation, original archive file is required to restore it (md5_err).
Exceptions	If errors are detected in a sample, the process can be reverted to verify the full batch.

**Table 8.** Check duplicates sub-task.

Agent	check-dups (script).
Function	Verify barcodes in a new batch against the ones already in the archive database.
Dependencies	None.
Target(s)	Master images of TIFF set (Image Processing sub-task 6).
Criteria	Checking eventual duplicates is done by a script which verifies that the barcodes in the batch have not been already used by looking up in the archive database.
Success	The set does not contain duplicate images (unique).
Fail	The set contain duplicate images which need to be further analysed to determine if they are valid duplicates or need to be flagged for removal from the set (duplicate).
Exceptions	Some types of duplicates are allowed, but require the intervention of a human operator.

**Table 9.** Check structure sub-task.

Agent	check-jp2-and-size (script).
Function	Verify that the images conform to the standards selected by MBG for long-term storage (TIFF) and high-definition production images (JP2).
Dependencies	JHOVE for analysing and checking that the images are well-formed (consistent with the basic requirements of the format) and valid (http://jhove.openpreservation.org/). Jpylyzer verifies if a JP2000 image really conforms to the format’s specifications (validation). It also reports the image’s technical characteristics (http://jpylyzer.openpreservation.org/).
Target(s)	Master images of TIFF set (Image Processing sub-task 7) production images on JPG and JP2 sets (Image Processing sub-task 7).
Criteria	TIFF images must conform to the TIFF 6.0 Specification. JP2000 images must conform to the JPEG 2000 image compression standard (ISO/IEC 15444-1).
Success	The image files conform to the corresponding standard (fss_ok).
Fail	The image files do not conform to the corresponding standard (fss_err).
Exceptions	Legacy scans prior to the implementation of the audit trail procedures may not conform to the current standards selected.

**Table 10.** Visual inspection sub-task.

Agent	Quality Manager (person).	
Function	Verify image quality by visually inspecting a sample of the images in the batch.	
Dependencies	Calibrated high pixel density display (e.g. Retina 5K Apple) Image editing programme (e.g. GIMP (The GIMP Team 2019) or Photoshop). Validation Checklist describing the steps of the inspection for selected images.	
Target(s)	Master images of TIFF set, only a subset is verified (Image Processing sub-task 8).	
Criteria	focus	Edges of the elements (specimen, labels, charts) are well defined, the text is readable.
	cropping	All elements of the specimen are visible in the image frame, i.e. no parts seem to extend beyond the edge of the image.
	exposure	Verify white balance using the white box of the colour chart and verify its average value: Above 250: Image overexposed -> reject Below 225: Image underexposed -> reject Verify black balance using the black box of the colour chart and verify its average value: Above 18: Image overexposed -> reject Below 12: Image underexposed -> reject The digitisation team established the limits by considering that, outside the colour chart, no details are visible if the level is lower than 12 for white or higher than 250 for black, these are equivalent to having 'holes' without data. The complementary values 225 and 18 are provided for reference.
	barcode	Verify that the name of the file is the same as the barcode on the sheet.
Success	Images meet visual quality criteria (vqc_ok).	
Fail	Images do not meet visual quality criteria (vqc_err). In this case, the operator needs to verifty another sample to determine if the whole batch should be rejected.	
Exceptions	Reference values need to be verified depending on the colour chart. usedSpecimens have been photographed with two types of colour chart: Standard CIE D50 Illuminant D50, Macbeth ColorChecker and ISA Golden Thread target.	

**Table 11.** Remove Duplicates Sub-task.

Agent	Quality Manager (person).
Function	Remove images flagged as bad crops or duplicates.
Dependencies	Error log report with list of non-compliant images.
Target(s)	Master images of TIFF set (Store Image sub-task 1). Production images on JPG and JP2 sets (Store Image sub-task 1).
Criteria	If an image in one of the sets is flagged (TIFF, JP2 or JPG), that image is removed from the set and all corresponding images in the other sets are also removed.
Success	Flagged images have been removed (dup_rmv).
Fail	Flagged images have been removed (dup_err).
Exceptions	If flagged images are part of the production set, the corresponding image from the master set needs to be validated to determine if the error was generated when the derivatives were produced or it is an imaging error.

**Table 12.** Check if production set stored sub-task.

Agent	check-if-archived (script).
Function	Verify that the production set images have been copied to the image repository and the back-up server.
Dependencies	Logs containing the paths to the servers where the image sets are stored. Read access to server for verification of file paths.
Target(s)	Production images on JPG and JP2 sets (Store Image sub-task 6).
Criteria	File paths for the images in the production sets need to be valid and non-empty.
Success	Image files are stored and backed up (stgv_ok).
Fail	Error in the image files store/back up process (stgv_err). Verify if storing procedure was performed and terminated with no errors.

**Table 13.** Check if production set stored sub-task.

Agent	del-dir-viaa (script).
Function	Delete master set copy from the buffer server, once reception and archiving is confirmed.
Dependencies	Confirmation from contractor of archiving of TIFF set.
Target(s)	Master images of TIFF set (Store Image subtask 7).
Criteria	The acknowledge code from contractor indicates that the master set has been received and archived.
Success	The image files are archived and the buffer has been cleared (bufc_ok).
Fail	Archiving of image files is not confirmed (bufc_err). Verify if copy files to archive task was performed and terminated with no errors.
Exceptions	Retry copy files to archive sub-task.

## Footnotes

*1 Images obtained from MBG data portal: http://www.botanicalcollections.be/specimen/BR0000013305871 and http://www.botanicalcollections.be/#/en/details/BR0000010148600

*2 As stated above, an image set consists of at least three images per specimen: Archive Master Image (TIFF), High-Definition Production Image (JP2) and Lightweight Publishing Image (JPG). If a specimen requires more than one image, for instance including booklets attached to herbarium sheets, the image set grows proportionally.

*3 Updated version of the workflow included in Picturae (2017)

*4 The commercial price is provided for reference, using the publicly avaliable storage pricing list for Amazon Web Services (Amazon S3) as advertised during December 2019 (https://aws.amazon.com/s3/pricing/). Prices are converted from United States Dollars (USD), using an exchange rate of 0.91. In addtition to storage prices, Amazon also charges a fees for data access requests, data transfer and management and replication which all add up to the total cost of storing data. Other providers, such as Google and Microsoft, offer similar pricing schemes for storage (i.e. storage + transaction + management costs which vary according to size and access type).
